# Effects of Repeated Smoking and Quitting Cigarettes on Plasma Concentrations of Clozapine and Its N‐Desmethyl and N‐Oxide Metabolites in a Japanese Patient With Schizophrenia

**DOI:** 10.1002/npr2.70121

**Published:** 2026-04-09

**Authors:** Kazuro Ikawa, Norifumi Morikawa, Mutsumi Sakata, Naoki Horikawa

**Affiliations:** ^1^ Graduate School of Biomedical and Health Sciences Hiroshima University Hiroshima Japan; ^2^ Nozoe Hills Hospital Kurume Japan

**Keywords:** clozapine, interaction, pharmacokinetics, smoking

## Abstract

Clozapine (CLZ) is used for treatment‐resistant schizophrenia. However, its pharmacokinetics in Japanese patients with schizophrenia have not been well described. Smoking affects the activities of cytochrome P450 (CYP), which mainly metabolizes CLZ, and patients with schizophrenia have a higher rate of smoking than others. Hence, the effects of smoking or quitting cigarettes on CLZ pharmacokinetics have been reported. Nevertheless, there are no case reports on interactions with CLZ, particularly examining OCLZ concentrations over time during repeated periods of smoking and quitting cigarettes. In a Japanese man in his 40s, trough plasma concentrations of CLZ, N‐desmethyl clozapine (NCLZ) and N‐oxide clozapine (OCLZ) were measured, and their ratios and the daily dose (D) were evaluated as indicators of drug metabolism. The CLZ/D ratio ([ng/mL]/[mg/day]) decreased by a median of 58.2%, whereas both NCLZ/CLZ and OCLZ/CLZ ratios increased by medians of 64.7% and 58.6%, respectively, in the total smoking period, relative to the total quitting period. The drug concentrations and their metabolic ratios fluctuated with smoking (six cigarettes daily) on and off multiple times. These results suggest that the CYP1A2‐inducing effect of smoking on CLZ metabolism can be reproducible and emphasize the need for careful attention to smoking status changes, CLZ concentration measurement and dose checking in patients with schizophrenia who have a smoking habit. Further studies are required to confirm the pharmacokinetic findings obtained from the limited data in only one patient.

## Abstract 2

In a Japanese patient with schizophrenia, the effects of repeated smoking and quitting cigarettes on plasma concentrations of clozapine (CLZ) and its major metabolites, N‐desmethyl clozapine (NCLZ) and N‐oxide clozapine (OCLZ), were shown to be reproducible.

## Graphical abstract

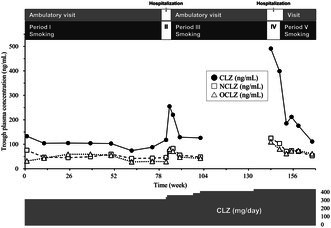

## 1 Introduction

Clozapine (CLZ) is an atypical antipsychotic drug used for the treatment of treatment‐resistant schizophrenia [1]. In Japan, CLZ tablets have been clinically used since July 2009 [2]. As the concentration of CLZ has been shown to correlate with its efficacy and safety, the pharmacokinetics of CLZ in Japanese patients have not been fully characterized. CLZ is mainly metabolized by hepatic cytochrome P450 (CYP) and CYP‐mediated interactions can occur. Smoking affects the activities of CYP, particularly CYP1A2, [3] while patients with schizophrenia are known to have a higher rate of smoking than other populations [4, 5]. Therefore, the effects of smoking or quitting cigarettes on CLZ pharmacokinetics in patients with schizophrenia have been reported [6, 7, 8, 9, 10, 11]. Nevertheless, there are no case reports on pharmacokinetic interactions with CLZ over multiple periods of smoking and quitting cigarettes. Additionally, the effects of smoking or quitting cigarettes have usually been reported for unchanged CLZ, and not for its major metabolites, N‐desmethyl clozapine (NCLZ) and N‐oxide clozapine (OCLZ). The concentrations of these metabolites themselves have not been shown to have clinical significance in decision‐making. However, the concentration ratios of NCLZ/CLZ and OCLZ/CLZ have been indicated as a useful adjunct for interpreting metabolism primarily [12].

In this report, we present a Japanese patient with schizophrenia to examine how repetition of smoking and quitting cigarettes affects plasma concentrations of CLZ, NCLZ, and OCLZ, and their concentration ratios over time.

## 2 Materials and Methods

### 2.1 Measurement of Plasma Concentrations of CLZ, NCLZ, and OCLZ

Venous blood was collected before the next dose of CLZ (approximately 10–14 h after the last dose). Blood samples were centrifuged and trough concentrations were measured in the resulting plasma.

The plasma concentrations of CLZ, NCLZ, and OCLZ were simultaneously measured using reversed‐phase high‐performance liquid chromatography according to the methods of Avenoso et al. [13] with minor modifications. Briefly, plasma samples (600 μL) were deproteinized by adding NaOH (0.5 M) and hexane:3‐methyl‐1‐butanol (75:25 [v/v]) using triprolidine as an internal standard. The mixture was vortexed and centrifuged, and the resulting upper layer was added for extraction to phosphate buffer (0.1 M, pH 2.2) and diethyl ether. After vortexing and centrifugation, the bottom layer (20 μL) was injected into a chromatograph, separated on an analytical column (Spherisorb C_6_ Column, 5 μm, 4.6 × 250 mm; Waters Corporation, Milford, MA, USA) at 40°C, and detected by ultraviolet absorbance (254 nm). The mobile phase was phosphate buffer (0.06 M, pH 2.7) containing sodium 1‐heptanesulfonate:acetonitrile (70:30 [v/v]) at a flow rate of 1 mL/min. The lower limit of quantification was 10 ng/mL, and the calibration curves were linear up to 1500 ng/mL for CLZ, NCLZ, and OCLZ. In intra‐day and inter‐day assays, the accuracy (as absolute error from 100%) and precision (as coefficient of variation) values were within 10% for all analytes.

## 3 Case Presentation

A Japanese man in his 40s (height 173 cm, median weight 71.4 kg) was diagnosed with treatment‐resistant schizophrenia. He was treated with CLZ at low doses, which were minimally necessary for him to live in the community. Clonazepam (2 mg/day) was orally administered to treat agitation, aggression, and dysphoria, and nitrazepam (5 mg/day) was orally administered to treat insomnia, as both concomitant drugs may neither induce nor inhibit CYP activities [14, 15].

The patient was a smoker who wanted to quit smoking. As self‐reported information, he smoked paper cigarettes (six cigarettes daily) during ambulatory visits. During hospitalization (80–84 and 141–150 weeks), medical staff confirmed that he quit cigarettes. After discharge, he abandoned quitting cigarettes and resumed smoking at home. This behavior was repeated.

**FIGURE 1:**
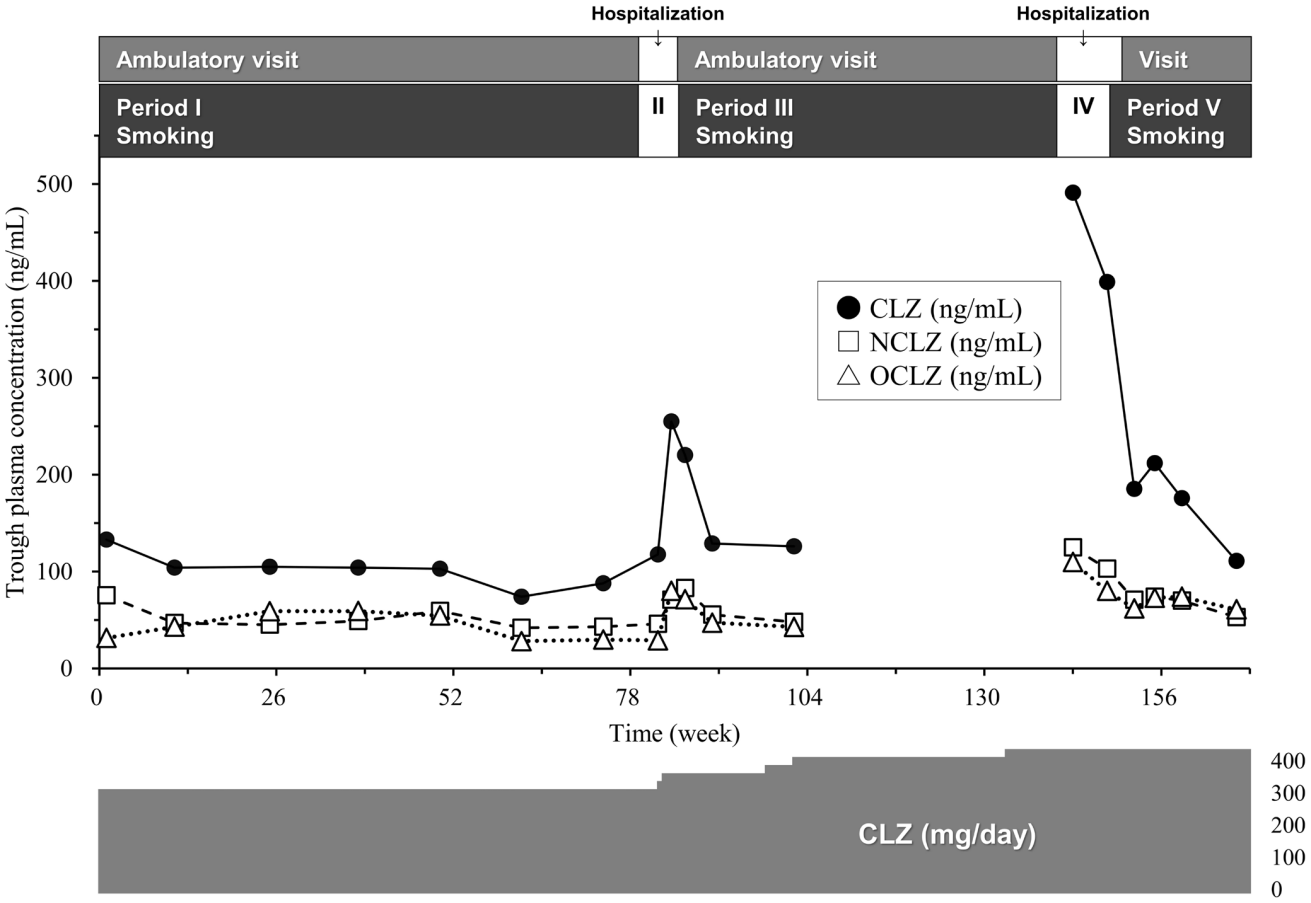
Trough plasma concentrations of clozapine (CLZ), N‐desmethyl clozapine (NCLZ), and N‐oxide clozapine (OCLZ) in smoking (I, III, V) and quitting (II, IV) periods during ambulatory visits and hospitalization.

Figure 1 shows the changes in plasma concentrations of CLZ, NCLZ, and OCLZ at 1–79 weeks (period I, smoking), 80–84 weeks (period II, quitting), 85–140 weeks (period III, smoking), 141–148 weeks (period IV, quitting), and afterward (period V, smoking) at the daily dose of 300–425 mg/day. The median CLZ concentrations were 104 ng/mL (period I), 118 and 255 ng/mL (period II), 129 ng/mL (period III), 491 and 399 ng/mL (period IV), and 181 ng/mL (period V). The drug concentrations fluctuated during these periods of smoking and quitting, although the CLZ concentrations were lower than the usual therapeutic range (350–600 ng/mL) [12, 16] because the doses were minimally required for his life in the community. Under these circumstances, values of laboratory tests such as hematological examination did not change by the daily dose increases. Assessment measures such as Brief Psychiatric Rating Scale were not used to precisely evaluate clinical response to CLZ.

Table 1 summarizes the ratios of the daily dose (D) and trough concentrations of CLZ, NCLZ, and OCLZ, as indicators of drug metabolism. The number of sampling points, particularly in each quitting period (II and IV), was too small to analyze. Therefore, the data were combined into the smoking or quitting period. The CLZ/D ratio ([ng/mL]/[mg/day]) decreased by a median of 58.2% (= 0.418‐fold lower) in the total smoking period (0.3485) relative to the total quitting period (0.834). The NCLZ/CLZ ratio increased by a median of 64.7% (= 1.647‐fold higher) in the total smoking period (0.4415) relative to the total quitting period (0.268). Likewise, the OCLZ/CLZ ratio increased by a median of 58.6% in the total smoking period (0.3735) relative to the total quitting period (0.2355).

**TABLE 1:** Ratios of the daily dose (D) and trough plasma concentrations of clozapine (CLZ), N‐desmethyl clozapine (NCLZ), and N‐oxide clozapine (OCLZ) in smoking (I, III, V) and quitting (II, IV) periods.

	Smoking period			Quitting period	
	Period I (7 points)	Period III (3 points)	Period V (4 points)	Period II (2 points)	Period IV (2 points)
CLZ/D ([ng/mL]/[mg/day])	[0.247, 0.347, 0.443]	[0.336, 0.369, 0.630]	[0.261, 0.425, 0.498]	0.392 and 0.729	1.16 and 0.939
	[0.247, 0.3485, 0.630] (14 points)			[0.392, 0.834, 1.16] (4 points)	
NCLZ/CLZ	[0.429, 0.460, 0.489]	[0.377, 0.381, 0.431]	[0.349, 0.3905, 0.477]	0.391 and 0.278	0.255 and 0.258
	[0.349, 0.4415, 0.489] (14 points)			[0.255, 0.268, 0.391] (4 points)	
OCLZ/CLZ	[0.236, 0.413, 0.570]	[0.324, 0.340, 0.367]	[0.335, 0.383, 0.550]	0.247 and 0.314	0.224 and 0.201
	[0.236, 0.3735, 0.570] (14 points)			[0.201, 0.2355, 0.314] (4 points)	

*Note:* [minimum, median (mean of the two medians for even number of points), maximum].

## 4 Discussion

We showed pharmacokinetic interactions with CLZ from smoking to quitting, and from quitting to smoking, and these behaviors were repeated multiple times. These interactions resulted in plasma concentration changes not only in the unchanged drug but also in its major metabolites. The concentrations and their metabolic ratios (CLZ/D, NCLZ/CLZ and OCLZ/CLZ) fluctuated in accordance with changes in smoking status. To the best of our knowledge, this is the first case report describing the effects of repeated smoking and quitting cigarettes on CLZ pharmacokinetics, particularly examining OCLZ concentrations over time in a patient with schizophrenia.

The CLZ/D ratio was lower in the smoking period than in the quitting period (Table 1). This result suggests that smoking decreases CLZ concentrations due to enhanced metabolism, which is consistent with many reports. With smoking, the CLZ/D ratio decreased on average: by 22.0% in 31 men and 54.8% in 13 women with 18 cigarettes daily [7]; by 38.6% in 38 men and 33 women [9]; and by 39.4% in 22 936 men and 44.7% in 10 393 women who smoked 16–20 cigarettes daily [11]. Conversely, with cessation of smoking, the CLZ/D ratio increased on average: by 33.2% in 13 men and 1 woman with ban of 7.3 cigarettes daily [8]; and by 43.2% in 13 men and 1 woman with cessation of 13.6 cigarettes daily [10]. Relative to these reported values for the smoking effect, the degrees of change in our patient (median reduction of 58.2%) were comparable with lower cigarette consumption (6 cigarettes daily). We consider this to be likely because Flanagan et al. [11] found the effect of smoking to achieve near maximal with 2–3 cigarettes daily in men and 4–5 cigarettes daily in women.

Plasma concentration after administration of CLZ was reported to be highest with unchanged CLZ, followed by the metabolites NCLZ and OCLZ (average ratio of CLZ:NCLZ:OCLZ = 1:0.59:0.19) [17]. The ratio of the major metabolite to CLZ was higher in the smoking period than in the quitting period (Table 1). This result is consistent with earlier reports. With smoking, the NCLZ/CLZ ratio increased on average: by 29.5% in 13 women [7]; by 25.9% in 38 men and 33 women [9]; and by 10.4% in 22 936 men and 14.5% in 10 393 women [11]. Conversely, with cessation of smoking, the NCLZ/CLZ ratio decreased on average: by 12.4% in 13 men and 1 woman [8]; and by 16.4% in 13 men and 1 woman in Japan [10].

Regarding the other metabolite, OCLZ is not well measured or analyzed in clinical pharmacokinetic studies, and there are no case reports about OCLZ in terms of smoking. The OCLZ/CLZ ratio was higher in the smoking period than in the quitting period (Table 1). In the metabolism from CLZ to NCLZ, its major contributors CYP1A2 and CYP3A4 were reported to account for approximately 30% and 22% of the metabolism, respectively [18]. From CLZ to OCLZ, CYP1A2 and CYP3A4 were reported to account for approximately 15% and 75%, respectively [19]. Smoking is considered to specifically induce CYP1A2 but may have only a minor inductive effect on CYP3A4 [3]. Hence, we consider it reasonable that smoking increased not only the NCLZ/CLZ ratio but also the OCLZ/CLZ ratio by enhancing CLZ metabolism mainly with a CYP1A2‐inducing effect.

The median CLZ/D ratios during periods I, II, III, IV and V were 0.347, 0.392, 0.729, 0.369, 1.16 and 0.939, and 0.425, respectively (Table 1). The corresponding values were 0.460, 0.391, 0.278, 0.381, 0.255 and 0.258, and 0.3905 for NCLZ/CLZ, and 0.413, 0.247, 0.314, 0.340, 0.224 and 0.201, and 0.383 for OCLZ/CLZ, respectively (Table 1). The fluctuations in these values suggest that the effect of smoking is reproducible, as the activities of CYP (particularly CYP1A2) were induced by smoking, then decreased and returned to the prior level with cessation of cigarettes, and increased again with smoking. Smoking rates were reported to be approximately twice as high in patients with schizophrenia as in other populations [4, 5]. Some patients with schizophrenia, such as our case, attempt to quit smoking for health or other reasons, but the addictive nature of nicotine often causes them to smoke cigarettes again. These behaviors alter drug metabolism, thereby causing fluctuations in the pharmacokinetics of CLZ. Therefore, clinicians and medical staff should pay careful attention to the current smoking status of patients with schizophrenia who have a smoking habit (especially outpatients), CLZ concentration measurement and dose checking. Additionally, observing NCLZ/CLZ and OCLZ/CLZ ratios can be useful to interpret not only CYP activity changes (by drug–drug interactions as well as cigarettes) but also drug adherence (levels of CLZ rise earlier than those of the metabolites when redosing after nonadherence), and clinical conditions such as inflammation and infection status (metabolism is suppressed by cytokines).

Finally, this case report has several limitations. The sample size was small, with only one patient, and the sampling points were small especially in quitting periods II and IV (2 points each). Also, these quitting periods almost coincided with hospitalization periods (Figure 1), hospital environment factors, such as regular hours life with controlled medication adherence and nutrition management, could have influenced the drug concentration changes. In addition, there was a long sampling interval between 102 and 143 weeks (before the particularly high CLZ concentration), which cannot exclude the possibility of changes in other factors. Moreover, sex and age differences in the effects of repeated smoking and quitting cigarettes have not been examined. Further investigations are needed to address these issues and to confirm the findings of this study.

## 5 Conclusion

The effects of repeated smoking and quitting cigarettes on plasma concentrations, not only of CLZ but also of its major metabolites NCLZ and OCLZ, were shown in a Japanese patient with schizophrenia. The drug concentrations and their metabolic ratios (CLZ/D, NCLZ/CLZ and OCLZ/CLZ) fluctuated in accordance with the CYP1A2‐inducing effect of smoking on and off. These results emphasize the need for careful attention to smoking status changes, CLZ concentration measurement and dose checking in patients with schizophrenia who have a smoking habit. Further studies are required to verify and generalize these pharmacokinetic findings obtained from the limited data in only one patient.

## Author Contributions

K.I. analyzed the plasma concentrations of CLZ, NCLZ, and OCLZ, and wrote the first draft of the manuscript. N.M. reviewed the pharmacokinetic data and refined the manuscript. M.S. and N.H. were involved in treating the patient and collecting and interpreting the data. All authors read, commented on, improved the manuscript, and approved the final version for submission.

## Funding

The authors have nothing to report.

## Ethics Statement

The study was approved by the Ethics Committee of Nozoe Hills Hospital, Kurume, Japan and performed in accordance with the Japanese Ethical Guidelines for Medical and Health Research Involving Human Subjects.

## Consent

The patient provided written informed consent.

## Conflicts of Interest

The authors declare no conflicts of interest.

## Data Availability

The essential data are presented in this report. In terms of ethical concerns, no detailed data are provided to protect personal information.
